# Quality of Pancreatic Neuroendocrine Tumor Videos Available on TikTok and Bilibili: Content Analysis

**DOI:** 10.2196/60033

**Published:** 2024-12-11

**Authors:** Zheyu Niu, Yijie Hao, Faji Yang, Qirong Jiang, Yupeng Jiang, Shizhe Zhang, Xie Song, Hong Chang, Xu Zhou, Huaqiang Zhu, Hengjun Gao, Jun Lu

**Affiliations:** 1 Department of Hepatobiliary Surgery, Shandong Provincial Hospital Affiliated to Shandong First Medical University, 324 Jingwuweiqi Road, Jinan, 250021, China, 86 13156109396; 2 Department of Clinical Research, Qilu Synva Pharmaceutical Company Limited, Dezhou, China; 3 Department of Physiology and Pathophysiology, School of Basic Medical Sciences, Cheeloo College of Medicine, Shandong University, Jinan, China; 4 Department of Clinical Research, Futaste Pharmaceutical Company Limited, Yucheng, China

**Keywords:** pancreatic neuroendocrine tumors, short videos, quality analysis, TikTok, Bilibili, social media

## Abstract

**Background:**

Disseminating disease knowledge through concise videos on various platforms is an innovative and efficient approach. However, it remains uncertain whether pancreatic neuroendocrine tumor (pNET)-related videos available on current short video platforms can effectively convey accurate and impactful information to the general public.

**Objective:**

Our study aims to extensively analyze the quality of pNET-related videos on TikTok and Bilibili, intending to enhance the development of pNET-related social media content to provide the general public with more comprehensive and suitable avenues for accessing pNET-related information.

**Methods:**

A total of 168 qualifying videos pertaining to pNETs were evaluated from the video-sharing platforms Bilibili and TikTok. Initially, the fundamental information conveyed in the videos was documented. Subsequently, we discerned the source and content type of each video. Following that, the Global Quality Scale (GQS) and modified DISCERN (mDISCERN) scale were employed to appraise the educational value and quality of each video. A comparative evaluation was conducted on the videos obtained from these two platforms.

**Results:**

The number of pNET-related videos saw a significant increase since 2020, with 9 videos in 2020, 19 videos in 2021, 29 videos in 2022, and 106 videos in 2023. There were no significant improvements in the mean GQS or mDISCERN scores from 2020 to 2023, which were 3.22 and 3.00 in 2020, 3.33 and 2.94 in 2021, 2.83 and 2.79 in 2022, and 2.78 and 2.94 in 2023, respectively. The average quality scores of the videos on Bilibili and Tiktok were comparable, with GQS and mDISCERN scores of 2.98 on Bilibili versus 2.77 on TikTok and 2.82 on Bilibili versus 3.05 on TikTok, respectively. The source and format of the videos remained independent factors affecting the two quality scores. Videos that were uploaded by professionals (hazard ratio=7.02, *P*=.002) and recorded in specialized popular science formats (hazard ratio=12.45, *P*<.001) tended to exhibit superior quality.

**Conclusions:**

This study demonstrates that the number of short videos on pNETs has increased in recent years, but video quality has not improved significantly. This comprehensive analysis shows that the source and format of videos are independent factors affecting video quality, which provides potential measures for improving the quality of short videos.

## Introduction

Pancreatic neuroendocrine tumors (pNETs) are heterogeneous neoplasms of the neuroendocrine system originating from the pancreatic islets‚ accounting for 3‐5% of all pancreatic malignancies [1,2]. Although relatively rare, the incidence of pNETs has been steadily increasing over recent decades, posing an increasing threat to public health [3]. Radical resection remains the most efficacious treatment for pNETs. Patients who undergo a radical resection typically exhibit a more favorable prognosis, with 5-year survival rates ranging from 65-86% [4]. However, patients with highly malignant pancreatic neuroendocrine carcinomas rarely survive beyond one year [5]. Similar to most malignancies, early diagnosis and prompt treatment are imperative in improving the prognosis of patients with pNETs [6]. However, the general populace, and even some health care professionals, possess inadequate knowledge about pNETs, hindering early detection and timely intervention to some extent. Hence, enhancing public awareness regarding pNETs represents an unmet necessity.

With the growing prominence of social media, it has become an increasingly influential way to disseminate information, allowing the general public to gain access to knowledge. In recent years, video content has gradually supplanted traditional text-based information as the primary medium for social media communication due to its vivid and efficient characteristics [7]. Consequently, video platforms have emerged as principal conduits for information dissemination. In China, TikTok and Bilibili stand out as the top two social media platforms for video content, with hundreds of millions of users [8]. Short videos can be effortlessly created and shared on TikTok and Bilibili, and users can interact with them through various means, such as comments, likes, and other interactive features, rendering these platforms immensely popular [9]. Hence, the dissemination of disease-related knowledge in the form of short videos on these platforms can potentially be more efficient and impactful than traditional methods. Nonetheless, it is worth noting that, due to the absence of a filtering system, videos uploaded to these platforms are prone to poor content quality and reliability, and some may even disseminate misleading information [10,11]. Indeed, while there exist pNET-related videos on TikTok and Bilibili that provide patients with accessible information to enhance their awareness of this disease, the quality of such videos remains uncertain. Therefore, the objective of this study was to conduct a meticulous analysis of the quality of pNET-related videos accessible on TikTok and Bilibili.

By employing a comprehensive quality analysis approach, we can objectively evaluate the standard of these videos and contribute toward the formulation of targeted strategies for enhancing their quality [12-14]. Fortunately, there already exist evaluation systems like Global Quality Scale (GQS) and the modified DISCERN (mDISCERN) system, which have been used to appraise the content, quality, and reliability of short videos pertaining to various diseases on social media platforms. These systems offer us the suitable tools to delve deeper into our understanding of these concise videos [7,8,13-16]. In light of this, our study endeavors to perform an extensive quality analysis of pNET-related videos available on TikTok and Bilibili, with the intention of augmenting the development of social media, so as to provide the general populace with more comprehensive and appropriate avenues to access and acquire knowledge about pNETs. Ultimately, our aim is to foster the early detection and prompt treatment of pNETs.

## Methods

### Ethical Considerations

This study did not involve the use of clinical datasets, biological specimens, or laboratory animals. All data from Bilibili and TikTok used in this analysis were publicly available and did not raise any privacy concerns. Furthermore, all data were deidentified, and neither individual users, videos, nor screenshots were identifiable in this article or its supplementary materials. Therefore, ethical review was not required.

### Data Collection

Videos were procured from two web-based platforms, specifically Bilibili [17] and TikTok [18], on January 16, 2024, employing the search term “pancreatic neuroendocrine tumors” in Chinese. After eliminating duplicates, advertisements, and irrelevant videos, the following parameters were documented and examined for all eligible videos: title, web address, number of likes, number of comments, number of shares, upload date, days since publication, video duration, video content, video source, and number of views (only available on Bilibili).

### Video Quality Assessments

Two widely adopted standardized scales, namely the GQS and the mDISCERN scale, were employed to evaluate the quality of the videos [19,20]. The GQS encompassed scores spanning from 1-5. Generally, scores of 1‐2 points were indicative of subpar quality, 3 points denoted moderate quality, and 4‐5 points indicated high quality within the GQS framework. On the other hand, the mDISCERN system comprised 5 questions, with each affirmative answer warranting a score of 1, while each negative response carried a score of 0. In detail, the 5 questions determined whether: (1) the video was lucid, concise, and comprehensible; (2) the sources of information were reliable; (3) the information presented was balanced and unbiased; (4) additional sources of information were provided for patient consultation; and (5) areas of ambiguity or controversy were appropriately addressed. Ultimately, higher scores signified superior quality and reliability. Two investigators (YH and FY) independently reviewed and assessed the aforementioned videos. When any discrepancies or disagreements arose between the two investigators, two authors (ZN and HG) deliberated and established a mutually agreed-upon conclusion.

### Statistical Analysis

Continuous variables were portrayed using mean and SD or median and IQR, while categorical variables were expressed as frequencies and percentages. The Wilcoxon rank-sum test was employed to compare video parameters between Bilibili and TikTok for continuous variables. For the comparison of GQS and mDISCERN scores across different groups, an independent 2-tailed sample *t* test was used. The receiver operator characteristics curve was employed to appraise the performance of the predictive model. Spearman correlation analysis was conducted to evaluate the correlations. Univariate and multivariate logistic regression models were used to assess the associations between video scores and key variables, estimating odds ratios and 95% CIs. Variables with *P*<.05 were selected for inclusion in the multivariate analysis. All statistical tests were 2-sided, with statistical significance considered at a *P* value of <.05. All the aforementioned statistical analyses were performed using R version 4.2.1 (R Core Team).

## Results

### Overview of the Video Screening Process

**Figure 1.**
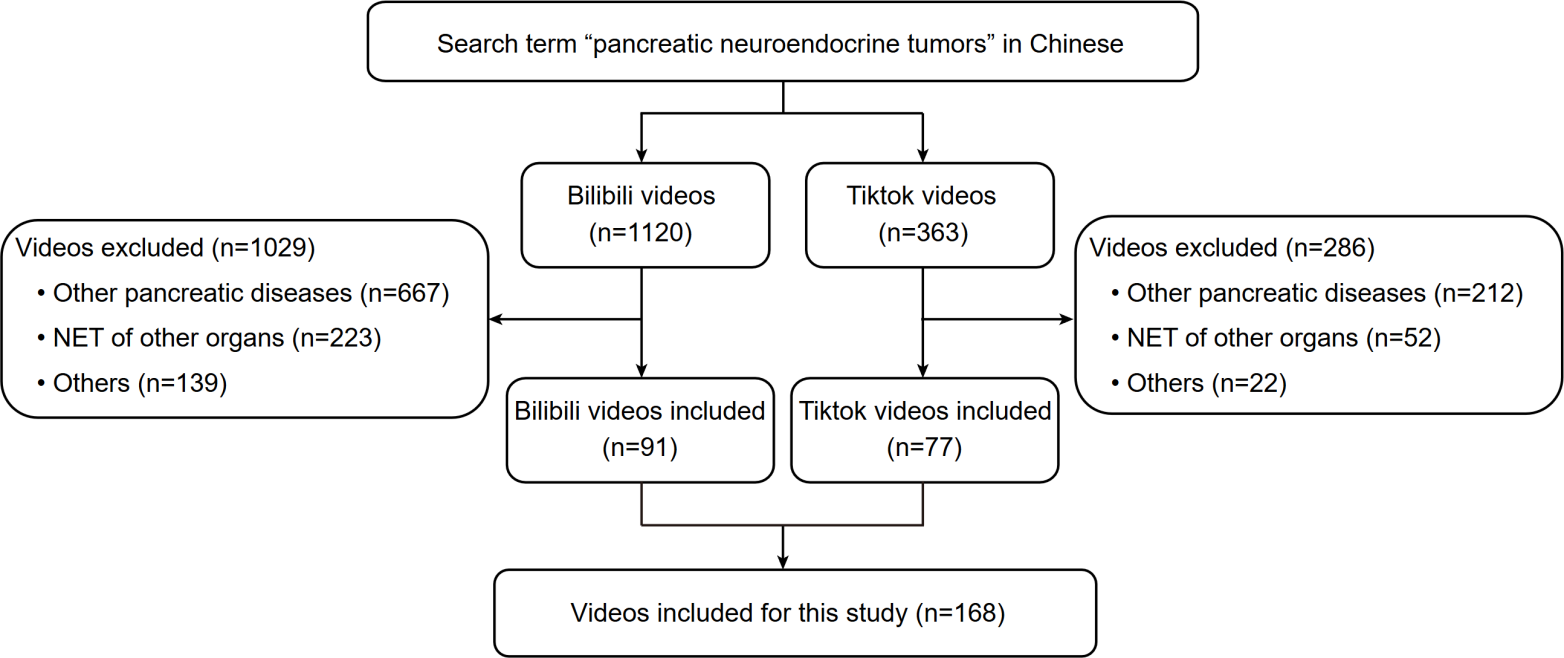
Flowchart for filtering pancreatic neuroendocrine tumor videos for analysis. NET: neuroendocrine tumor.

A total of 1120 and 363 videos pertaining to pNETs were retrieved from the web-based platforms of Bilibili and TikTok, respectively. Following a meticulous screening process, we successfully purged 1029 unrelated videos from Bilibili and 286 unrelated videos from TikTok. Consequently, a refined collection of 91 suitable videos from Bilibili and 77 videos from TikTok were included for the subsequent analysis. Ultimately, a comprehensive evaluation was conducted on an assortment of 168 eligible videos encompassing both the Bilibili and TikTok platforms (Figure 1).

### Landscape of the pNET-Related Videos on Tiktok and Bilibili

**Figure 2.**
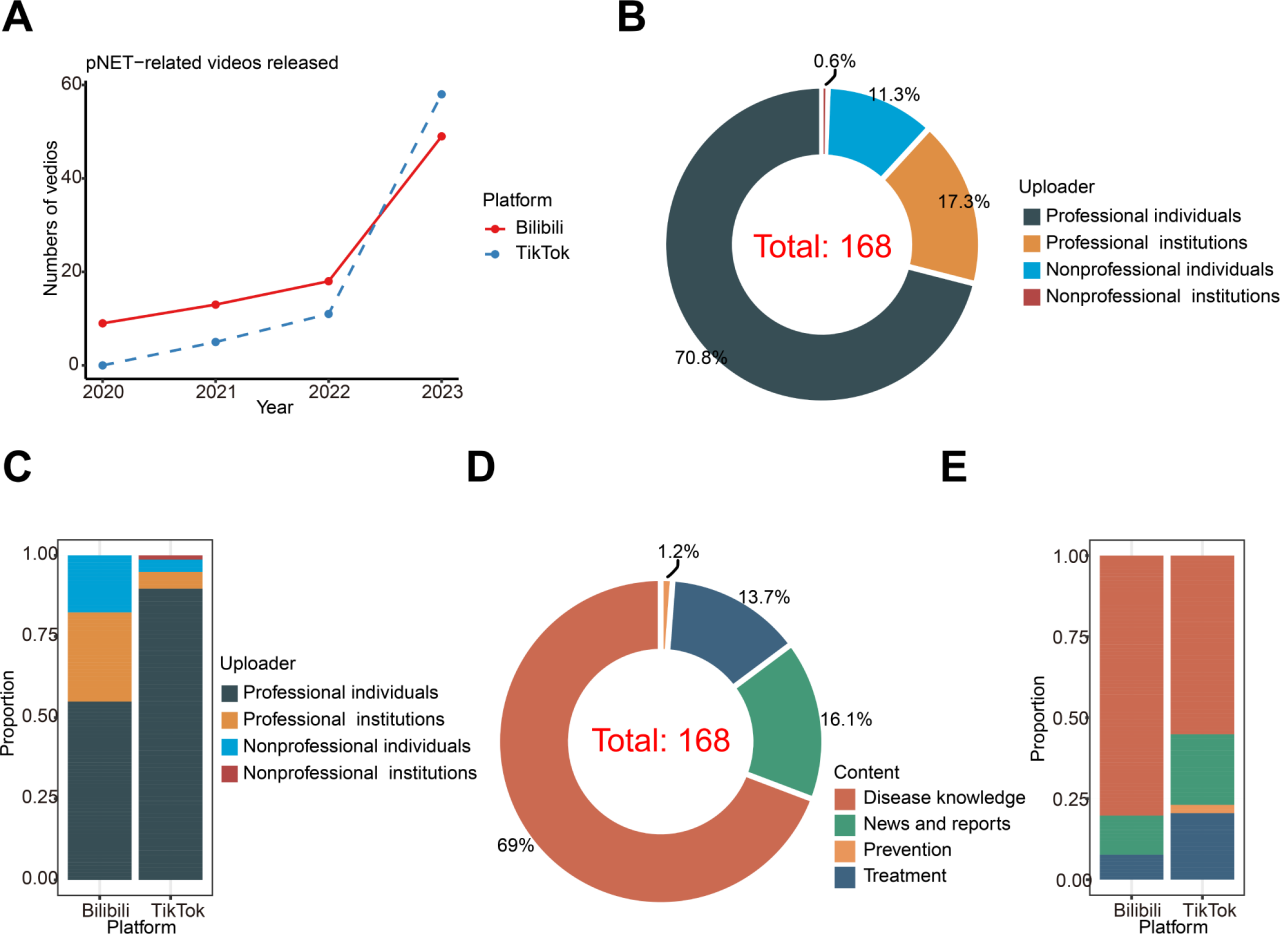
General information on pNET-related videos from Tiktok and Bilibili. (**A**) A line chart shows eligible pNET-related videos released between 2020 and 2023. (**B**) Doughnut chart shows the sources of all included videos. (**C**) Bar chart shows the sources of videos from Tiktok and Bilibili. (**D**) Doughnut chart shows the content of all included videos. (**E**) Bar chart shows the content of videos from Tiktok and Bilibili. pNET: pancreatic neuroendocrine tumor.

Following a comprehensive search, we discovered that there were no pNET-related videos on these two platforms prior to 2020, probably due to the platforms only becoming well-known in last several years. Specifically, of the 91 videos uploaded to Bilibili from 2020 to 2023, there were 9 videos (10%) in 2020, 13 videos (14%) in 2021, 18 videos (21%) in 2022, and 49 videos (53%) in 2023 (Figure 2A). On the other hand, of the 77 videos uploaded to TikTok, there were 5 videos (6%) in 2021, 11 videos (14%) in 2022, and 57 videos (74%) in 2023 (Figure 2A). Overall, the number of pNET-related short videos on Tiktok and Bilibili were similar, having increased sharply in recent years. In addition, our search period extended two weeks into 2024, during which 6 pNET-related videos in total were already present on TikTok and Bilibili (Multimedia Appendix 1).

Given the unrestricted nature of video uploads on these platforms, a diverse range of users contributed relevant content. In our study, uploaders were categorized as professional individuals, nonprofessional individuals, professional institutions, and nonprofessional institutions, based on their professionalism and whether they were operating as individuals or institutions. Statistical analysis revealed that a majority of pNET-related videos (119/168, 70.8%) were uploaded by professional individuals. The remaining sources comprised professional institutions (29/168, 17.3%), nonprofessional individuals (19/168, 11.3%), and nonprofessional institutions (1/168, 0.6%; Figure 2B). In general, the quantity of pNET-related videos originating from reputable sources on both TikTok and Bilibili greatly surpassed the quantity of videos from nonprofessional sources (Figure 2C). In terms of video content, disease knowledge predominated, accounting for 69% of content (116/168; Figure 2D). This trend held true for both TikTok and Bilibili, with professional individuals contributing the largest number of videos focused primarily on disease knowledge (Figure 2E).

### General Information and Index of Videos

The number of views, likes, comments, shares, and saves of the videos on TikTok and Bilibili is as an indicator for the attention received by these videos, making it an appropriate tool for evaluating their influence. First, we compared these indices between TikTok and Bilibili. On average, pNET-related videos on Bilibili accumulated around 291 views, with likes, comments, shares, and saves being relatively scarce (Table 1). Conversely, pNET-related videos on TikTok garnered frequent likes, comments, shares, and saves, significantly surpassing those on Bilibili (Table 1). However, the videos on Bilibili had longer durations and were published earlier. In summary, Bilibili featured longer videos released at earlier dates, while TikTok offered more interactive content (Table 1).

Next, we further compared the number of views, likes, comments, shares, and saves of the videos based on their source and content type. It was observed that videos uploaded by nonprofessional individuals attracted more attention on both Bilibili and TikTok, while those uploaded by professional individuals and institutions were less popular (Multimedia Appendix 2). When analyzing content types, treatment-related videos received less attention on both platforms, whereas videos related to news and reports received more attention. A difference between Bilibili and Tiktok was that videos related to disease knowledge were popular on Bilibili, but not on Tiktok (Table 2).

**Table 1.** Basic index of videos about pancreatic neuroendocrine tumors on Bilibili and TikTok.

Variable	Bilibili (n=91), median (IQR)	TikTok (n=77), median (IQR)	*P* value^a^
Likes	3.5 (1.0-13.75)	169.0 (62.8-459.3)	<.001
Comments	0 (0-0）	19.0 (6.0-89.3）	<.001
Shares	1.0 (0-7.8）	20.0 (5.0-67.5）	<.001
Saves	3.0 (0-29.5）	31.5 (8.0-105.8）	<.001
Days since published	309.5 (87.5-692.8）	141.5 (53.0-328.5）	<.001
Duration (s)	300.5 (109.0-1332.0）	55.0 (45.0-81.3）	<.001
Views	291.5 (125.8-708.8）	—^b^	—

a Wilcoxon rank-sum test was used.

b Views were not available on Tiktok.

**Table 2.** Comparison of source and content of videos about pancreatic neuroendocrine tumors on two platforms.

	Views	Likes	Comments	Saves	Shares	Days	Duration (s)
Bilibili, median (IQR)							
Nonprofessional individuals (n=16)	610.0 (255.5-2333)	12.0 (3.0-151.5)	0.0 (0.0-8.0)	12.0 (1.5-51.0)	3.0 (0.5-12.5)	478.0 (305.0-741.0)	235.0 (136.0-734.5)
Professional individuals (n=50)	300.5 (148.0-617.0)	4.0 (1.0-13.5)	0.0 (0.0-0.0)	6.0 (0.75-31.25)	1.0 (0.0-7.0)	318.0 (129.5-656.8)	495.5 (129.3-1737.0)
Professional institutions (n=25)	114.0 (84.0-789.5)	0.0 (0.0-6.5)	0.0 (0.0-0.0)	0.0 (0.0-13.5)	1.0 (0.0-7.5)	85.0 (57.5-1041.0)	153.0 (82.0-791.0)
Disease knowledge (n=73)	315.0 (128.0-702.0)	4.0 (1.0-13.0)	0.0 (0.0-0.0)	4.0 (0.75-28.5)	1.0 (0.0-8.3)	363.5 (129.5-753.0)	401.5 (142.8-1737.0)
News and reports (n=11)	261.0 (95.0-3390.0)	2.0 (0.0-278.0)	0.0 (0.0-5.0)	0.0 (0.0-36.0)	1.0 (0.0-6.0)	176.0 (57.0-478.0)	70.0 (43.0-207.0)
Treatment (n=7)	146.0 (42.0-629.0)	1.0 (0.0-15.0)	0.0 (0.0-0.0)	0.0 (0.0-25.0)	1.0 (0.0-15.0)	154.0 (85.0-317.0)	103.0 (60.0-669.0)
Tiktok, median (IQR)							
Nonprofessional institutions (n=1)	—^a^	1477.0 (1477.0-1477.0)	15.0 (15.0-15.0)	68.0 (68.0-68.0)	66.0 (66.0-66.0)	767.0 (767.0-767.0)	9.0 (9.0-9.0)
Nonprofessional individuals (n=3)	—	1109.0 (717.0-14000.0)	157.0 (120.0-2132.0)	310.0 (51.0-910.0)	286.0 (17.0-298.0)	292.0 (60.0-302.0)	19.0 (16.0-298.0)
Professional individuals (n=69)	—	158.5 (62.8-366.0)	17.5 (6.0-68.75)	26.5 (8.0-105.8)	18.0 (4.75-65.5)	125.0 (47.8-295.8)	55.0 (45.8-81.3)
Professional institutions (n=4)	—	104.0 (25.5-804.3)	15.0 (4.3-25.0)	32.5 (8.75-42.8)	43.0 (11.3-89.0)	701.5 (363.8-893.0)	58.5 (38.8-237.3)
Disease knowledge (n=43)	—	109.5 (49.0-256.0)	11.5 (4.0-33.8)	15.5 (5.3-62.3)	10.0 (2.3-56.8)	108.0 (48.8-318.0)	62.0 (47.0-81.8)
News and reports (n=16)	—	484.5 (130.8-1385.0)	23.0 (12.0-169.0)	59.5 (12.5-263.0)	24.5 (9.3-96.5)	260.0 (80.8-299.5)	47.0 (32.0-87.5)
Prevention (n=2)	—	676.5 (45.0-1308.0)	51.5 (4.0-99.0)	253.0 (4.0-502.0)	801.5 (1.0-1602.0)	359.0 (82.0-636.0)	49.0 (46.0-52.0)
Treatment (n=16)	—	360.5 (173.0-891.3)	35.0 (16.3-98.8)	59.0 (24.3-133.0)	45.0 (28.8-72.8.0)	185.0 (33.0-361.8)	55.0 (34.3-75.0)

a Views were not available on Tiktok.

### Quality Analysis of Web-Based Videos

**Figure 3.**
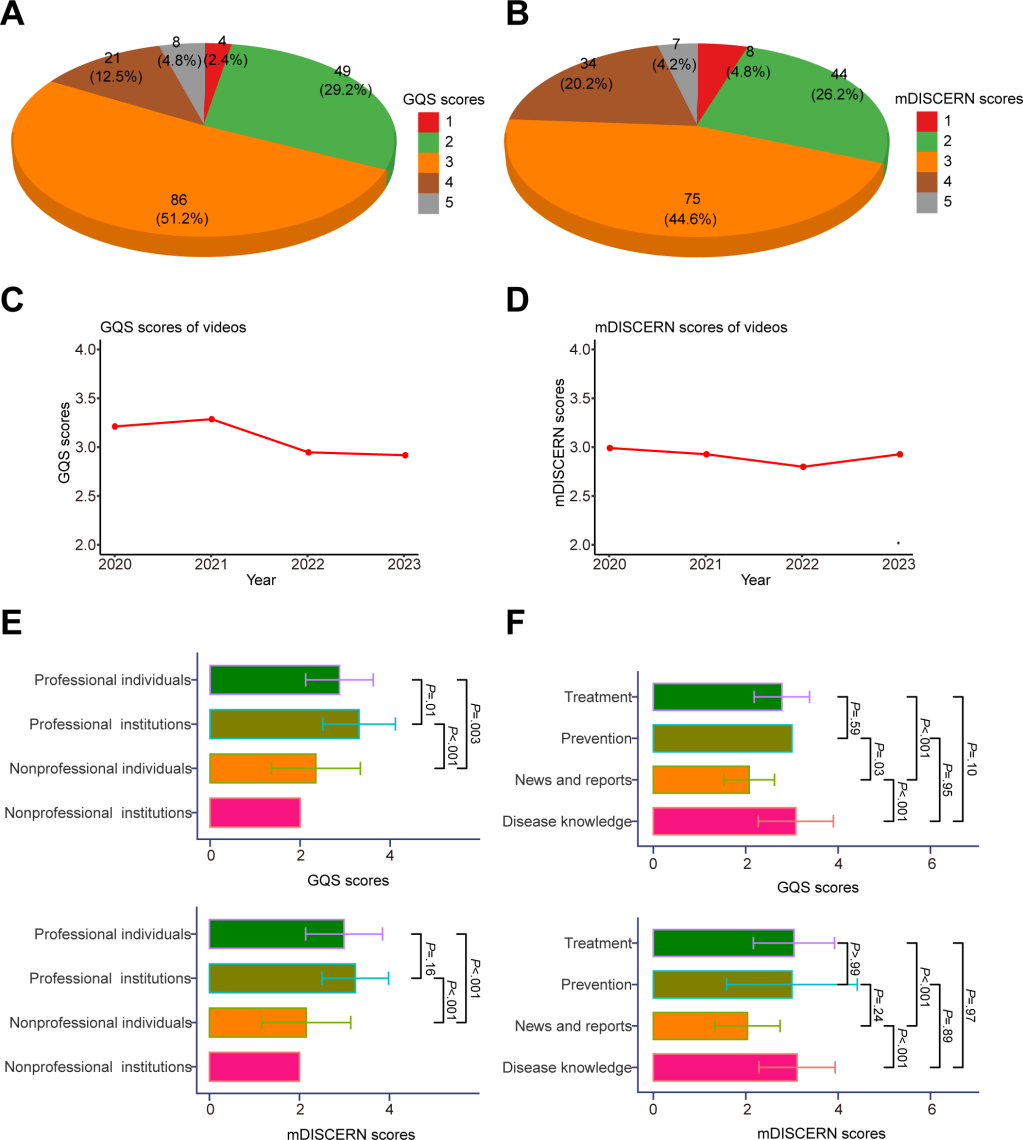
Quality analysis of videos. (**A**) A pie chart shows the GQS scores of all included videos. (**B**) A pie chart shows the mDISCERN scores of all included videos. (**C**) A line chart shows the mean GQS scores of eligible videos released between 2020 and 2023. (**D**) A line chart shows the mean mDISCERN scores of eligible videos released between 2020 and 2023. (**E**) Bar chart shows the GQS and mDISCERN scores of videos from different sources. (**F**) Bar chart shows the GQS and mDISCERN scores of videos according to the content. Error bars in panels E and F represent the SD from the mean, indicating the variability of the data. GQS: Global Quality Scale; mDISCERN: modified DISCERN.

In order to impartially evaluate the quality of the videos, we conducted a comprehensive analysis using the internationally recognized GQS and the mDISCERN scoring systems. Specifically for GQS scores, the majority of videos obtained a score of 3 (86/168, 51.2%), followed by scores of 2 (49/168, 29.2%), 4 (21/168, 12.5%), 5 (8/168, 4.8%), and 1 (4/168, 2.4%; Figure 3A). Similarly, in terms of mDISCERN scores, videos that were rated 3 accounted for the largest proportion at 44.6% (75/168). The remaining scores were as follows: 2 (44/168, 26.2%), 4 (34/168, 20.2%), 1 (8/168, 4.8%), and 5 (7/168, 4.2%; Figure 3B). These findings indicated that the majority of videos fell within the medium quality range, while the proportions of excellent and poor quality videos were small.

Subsequently, we analyzed the quality scores of the uploaded videos for each year spanning from 2020 to 2023, with the goal of investigating any gradual improvement in quality over recent years. The results revealed that the mean GQS scores were 3.22 in 2020, 3.33 in 2021, 2.83 in 2022, and 2.78 in 2023 (Figure 3C). Regarding mDISCERN scores, the mean scores were 3.00 in 2020, 2.94 in 2021, 2.79 in 2022, and 2.94 in 2023 (Figure 3D). The quality of pNET-related videos did not exhibited improvement in recent years, with slight declines observed in the GQS scores (Multimedia Appendix 3).

To explore whether the different types of uploaders had an impact on video quality, we further compared the GQS and mDISCERN scores among the different uploaders. It was observed that the ratings of GQS and mDISCERN scores were consistent. As anticipated, videos posted by professional individuals and institutions received higher scores (Figure 3E). Moreover, there were disparities in the quality ratings for different types of video content. Generally, the quality of videos related to news and reports was inferior, whereas the quality scores were similar for other video types (Figure 3F).

### Quality Comparison Across Platforms and Formats

**Figure 4.**
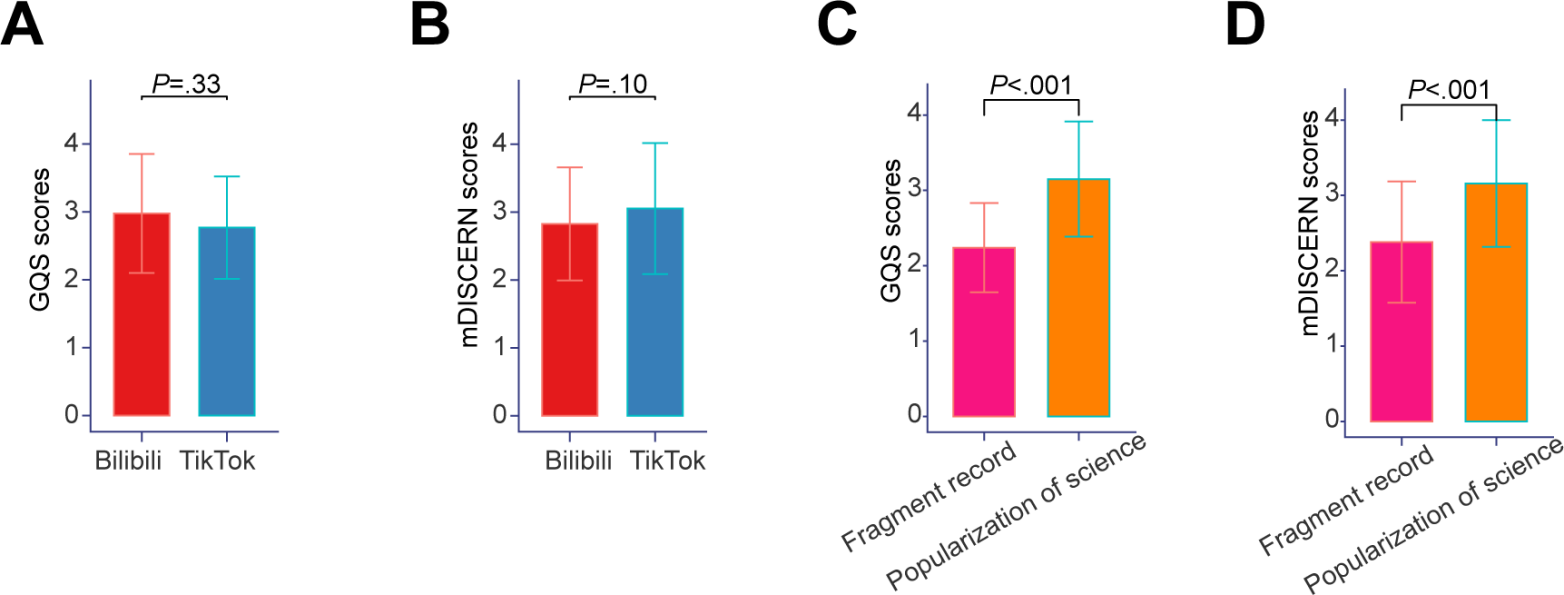
Quality comparison across platforms and formats. (**A**) A bar chart shows the mean GQS scores of videos from Tiktok and Bilibili. (**B**) A bar chart shows the mean mDISCERN scores of videos from Tiktok and Bilibili. (**C**) A bar chart shows the mean GQS scores of videos according to the the format. (**D**) A bar chart shows the mean mDISCERN scores of videos according to the the format. Error bars represent the SD from the mean. GQS: Global Quality Scale; mDISCERN: modified DISCERN.

Given the distinctive characteristics of Bilibili and Tiktok, we conducted a comparative analysis of video quality between these two platforms. The results indicated no significant difference in the GQS and mDISCERN scores (Figure 4A and B). For Bilibili, pNET-related videos possessed an average rating of 2.98 points on the GQS and 2.82 points on the mDISCERN scale. Similarly, for Tiktok, the average scores were 2.77 points on the GQS and 3.05 points on the mDISCERN scale. Notably, significant differences in quality were observed across different video formats. Specifically, all included videos could be classified into two categories: videos for the popularization of science with distinct themes and videos of fragments from a medical visit or meeting. The average GQS scores of videos for the popularization of science (3.15 points) were significantly higher than those of fragments of medical visits or meetings (2.24 points; Figure 4C). This trend was consistent with the average mDISCERN scores (3.15 points versus 2.38 points), which also indicated superior quality of videos for the popularization of science compared to those of medical visits or meetings (Figure 4D, Multimedia Appendix 4). Both the GQS and mDISCERN scores demonstrated that videos for the popularization of science exhibited superior quality compared to videos in the form of fragments from a medical visit or meeting.

### An Investigation Into Determinants of Video Quality

**Figure 5.**
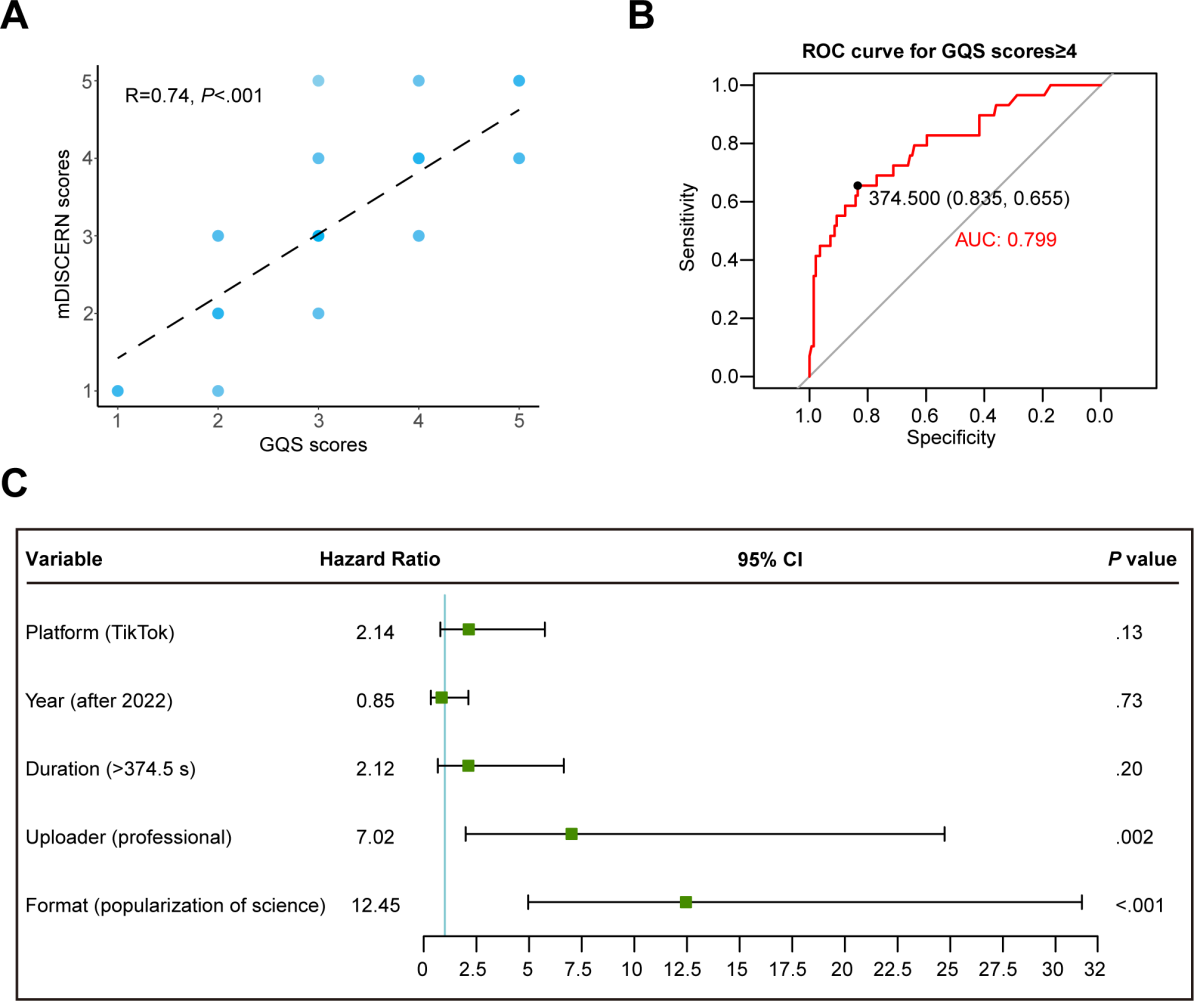
An investigation into determinants of video quality from Tiktok and Bilibili. (**A**) Correlation analysis between GQS scores and mDISCERN scores. The depth of blue represents the strength of the correlation between them. (**B**) Receiver operator characteristic curve of the video durations for GQS score prediction. The cutoff value (specificity, sensitivity) are shown in the figure. (**C**) Multivariate logistic analysis of potential determinants with GQS scores of ≥3. AUC: area under the curve; GQS: Global Quality Scale; mDISCERN: modified DISCERN; ROC: receiver operator characteristic.

Initially, a correlation analysis between the GQS scores and mDISCERN scores substantiated their congruity, validating the robustness of our scoring outcomes (Figure 5A). Subsequently, employing Spearman correlation analysis, we identified the connections between video variables and quality scores. Our findings indicated that both GQS scores and mDISCERN scores exhibited an exclusive correlation with the duration of the videos, while remaining unrelated to other factors, such as likes, comments, saves, shares, and days since the video was published (Multimedia Appendix 5). Moreover, the receiver operator characteristic curve revealed that the majority of videos with GQS scores and mDISCERN scores surpassing 4 points were longer than 374.5 seconds (Figure 5B, Multimedia Appendix 6).

Finally, a comprehensive analysis encompassing all potential factors influencing video quality was undertaken. The video duration, uploader, and format were identified as the prominent determinants significantly impacting video quality (Multimedia Appendices 7 and 8). In addition, a multivariate analysis revealed that only the uploader and format remained independent factors affecting video quality (Figure 5C, Multimedia Appendix 9). Put succinctly, videos that were uploaded by professionals and recorded in specialized popular science formats tended to exhibit superior quality.

## Discussion

### Principal Results

The management of neuroendocrine tumors, particularly of pancreatic origin, has drastically improved recently due to the establishment of neuroendocrine tumor–centralized facilities backed by societies like the European Neuroendocrine Tumor Society [21]. These centers provide a broader diagnostic evaluation crucial to effective therapy. They allow for the precise determination of the neuroendocrine tumor types and dimensions, aiding in the development of targeted treatments. Therapeutic approaches for pNETs necessitate, for the most part, prolonged adherence. Therefore, enhancing patient education to boost compliance is paramount to therapeutic efficacy. However, it can be challenging and confusing for patients to access specialized cutting-edge guidelines. Educational videos on social media provide an accessible platform for acquiring pNET-related knowledge.

Social media, with its extensive user base, swift dissemination of content, and automatic recommendation of relevant videos, has emerged as a beneficial health education platform. Its significant convenience for accessing comprehensive information on certain diseases has been acknowledged [22]. Research on stomach cancer revealed that scientific videos emphasize the importance of adopting preventative measures by altering lifestyles and implementing specific actions (eg, eradicating *Helicobacter pylori*), offering substantial benefits to patients [23]. Yet, research on breast cancer underscores the inadequacy of TikTok and Bilibili’s related health content [8]. However, videos that execute disease education, prevention, or treatment under expert guidance exhibit significant advantages. It is encouraged for medical professionals to proactively produce quality videos, augmenting their responsibility in health-video oversight and ensuring public access to reliable health care information [8]. A higher quality of videos uploaded by professionals and those with a popular science format have been observed for other diseases, such as osteosarcoma [24], liver disease [25], chronic kidney disease [26] and chronic obstructive pulmonary disease [27]. This is attributed to the diligent efforts of clinicians and scientific writers in providing trustworthy and comprehensible information.

Given the scarce and obscure symptoms of pNETs, late detection often occurs. The disease requires complex and continually evolving treatment methods, often leading to hesitation and consequent negative impacts on the treatment outcome due to lengthy processes, frequent regimen modifications, and financial stress. High-quality social media videos can assist in enhancing patient understanding and improving treatment compliance. However, social media carries potential risks, as it can distribute erroneous and provocative information just as rapidly. We discovered during video screening that many merchants posing as professionals promote unverified and unreliable treatments under the guise of popular science, necessitating caution.

Our research reveals that on Bilibili and Tiktok, the most frequently used video social media platforms among Chinese users, pNET-related posts were only discoverable after 2020. This signifies that Chinese societal awareness of this disease emerged relatively recently. Analysis of these two platforms shows that the majority of video producers are professionals or institutions, with fewer general science writers involved, reflecting a knowledge gap in pNET promotion. However, the volume of pNET videos is increasing annually, suggesting positive trends with more professional posts and increased attention.

The GQS and improved mDISCERN assessment scores for pNET content yielded results of 2.88/5 and 2.93/5, respectively, indicating a subpar performance across the board. Extended professional videos bored viewers while short ones struggled to meet high quality standards due to having a limited scope. Furthermore, the low barrier to entry for video creators and the potential dissemination of erroneous information pose challenges on social media for fact-checking by audiences. Therefore, significant strides are needed to elevate the quality and appeal of pNET video content. This necessitates the involvement of professionals such as clinicians in creating science-based videos, alongside robust quality control procedures from platform stakeholders. Considerations like establishing dedicated medical video categories, incorporating precautionary intros, and having a scientific review team audit these videos are needed. Patients must also actively discern reliable sources and consult doctors rather than depend solely on social media content.

### Limitations

This study’s limitations must be explicitly acknowledged. Only Chinese videos within China were considered, with YouTube, the predominant global short video platform, excluded due to its restricted accessibility for ordinary Chinese citizens. Given the limited English proficiency among Chinese patients, relevant English content was also ruled out. Furthermore, the ability of video uploaders to remove content could introduce bias into the search results.

### Conclusions

In summary, to our knowledge, this is the first study to investigate the quality of pNET-related videos on short video platforms. The study demonstrated that the number of pNET-related videos has increased dramatically in recent years, but the video quality has not improved significantly. The findings of this study provided potential measures for improving the quality of short videos.

## Supplementary material

10.2196/60033 Multimedia Appendix 1 Video information.

10.2196/60033 Multimedia Appendix 2 Comparison of popularity between professionals and nonprofessionals on two platforms.

10.2196/60033 Multimedia Appendix 3 Score line chart of pancreatic neuroendocrine tumor videos from 2020 to 2023.

10.2196/60033 Multimedia Appendix 4 Comparison of scores for videos in different formats on different platforms.

10.2196/60033 Multimedia Appendix 5 Correlation between video variables and quality scores.

10.2196/60033 Multimedia Appendix 6 Receiver operator characteristic curve of durations for modified DISCERN scores of ≥4 prediction.

10.2196/60033 Multimedia Appendix 7 Univariate analysis of risk factors for Global Quality Scale scores.

10.2196/60033 Multimedia Appendix 8 Univariate analysis of risk factors for modified DISCERN scores.

10.2196/60033 Multimedia Appendix 9 Multivariate logistic regression analysis of potential determinants with modified DISCERN scores of ≥3.

## Abbreviations

GQS: Global Quality Scale
mDISCERN: modified DISCERN
pNET: pancreatic neuroendocrine tumor
