# Supplementary material for: Quality of Pancreatic Neuroendocrine Tumor Videos Available on TikTok and Bilibili: Content Analysis
Source: JMIR Form Res. 2024 Dec 11;8:e60033. doi: 10.2196/60033 (PMC11655045; doi:10.2196/60033)
Supplement: Multimedia Appendix 2 [file formative-v8-e60033-s002.docx]

|  | Bilibili | | | Tiktok | | |  |
| --- | --- | --- | --- | --- | --- | --- | --- |
|  | Professional, N = 75^a^ | nonProfessional, N = 16^a^ | *P* value^b^ | Professional, N = 73^a^ | nonProfessional, N = 4^a^ | *P* value^b^ | |
| Views | 261.0 (112.0, 624.0) | 544.5 (285.3, 1,803.8) | .02 | / | / | / | |
| Likes | 3.0 (0.0, 11.5) | 13.5 (3.0, 88.3) | .009 | 156.0 (63.0, 365.0) | 1,293.0 (1,011.0, 4,607.8) | .007 | |
| Comments | 0.0 (0.0, 0.0) | 0.0 (0.0, 6.5) | .12 | 18.0 (6.0, 62.0) | 138.5 (93.8, 650.8) | .04 | |
| Saves | 2.0 (0.0, 25.5) | 18.5 (1.8, 43.5) | .07 | 31.0 (8.0, 94.0) | 189.0 (63.8, 460.0) | .03 | |
| Shares | 1.0 (0.0, 6.5) | 4.0 (0.8, 11.3) | .20 | 18.0 (5.0, 67.0) | 176.0 (53.8, 289.0) | .06 | |
| Days | 263.0 (84.5, 686.5) | 485.5 (311.00, 685.0) | .045 | 137.0 (50.0, 328.0) | 297.0 (234.0, 418.3) | .30 | |
| Duration (s) | 321.0 (106.0, 1,523.5) | 232.0 (146.0, 714.8) | .80 | 56.0 (46.0, 81.0) | 17.5 (14.3, 88.8) | .10 | |
| ^a^Median (IQR); ^b^Wilcoxon rank sum test. | | | | | | | |
