# Supplementary figures and images for: Quality of Pancreatic Neuroendocrine Tumor Videos Available on TikTok and Bilibili: Content Analysis
Source: JMIR Form Res. 2024 Dec 11;8:e60033. doi: 10.2196/60033 (PMC11655045; doi:10.2196/60033)

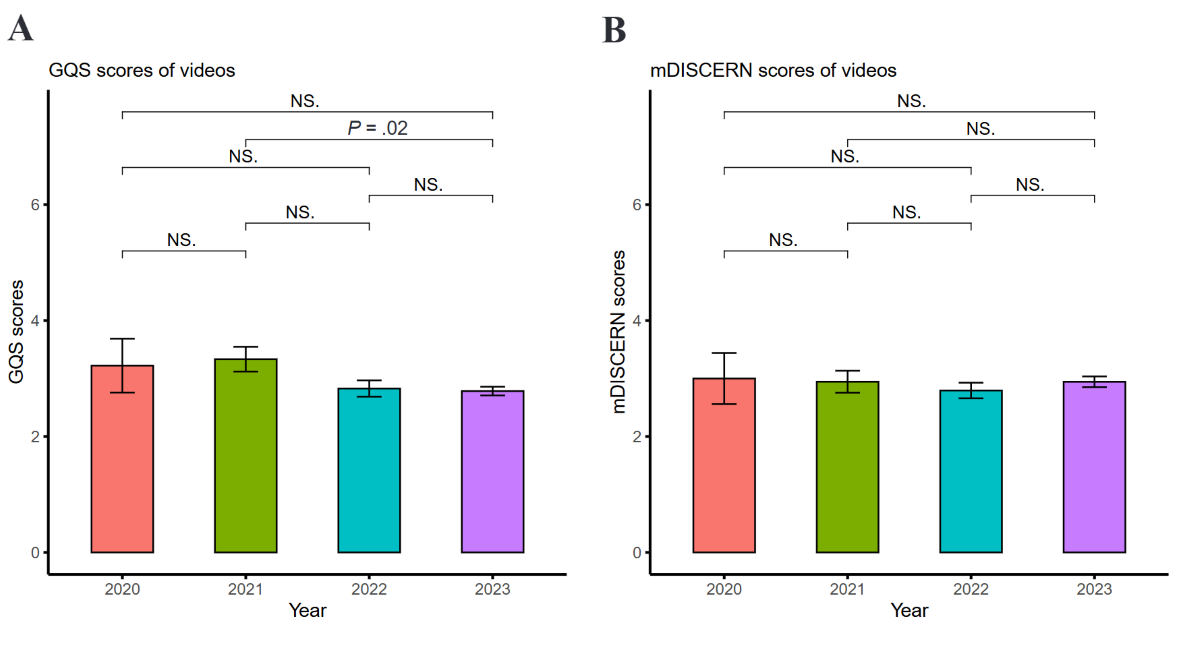

Supplement: Multimedia Appendix 3 [file formative-v8-e60033-s003.png]

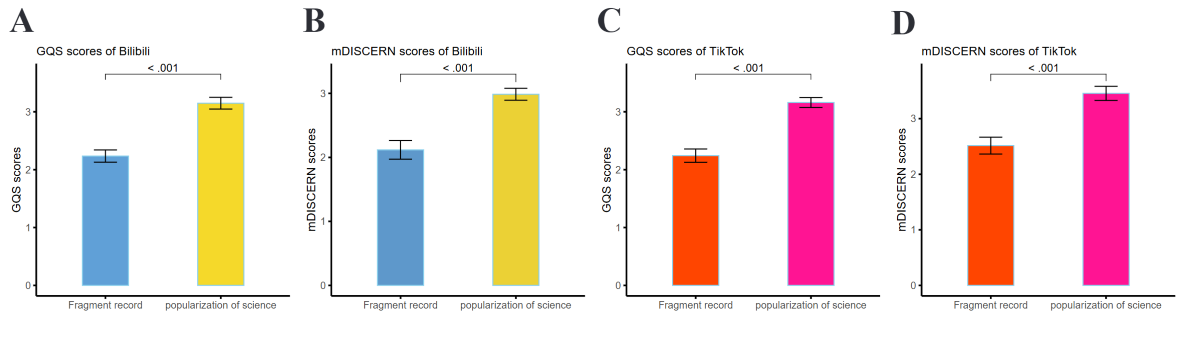

Supplement: Multimedia Appendix 4 [file formative-v8-e60033-s004.png]

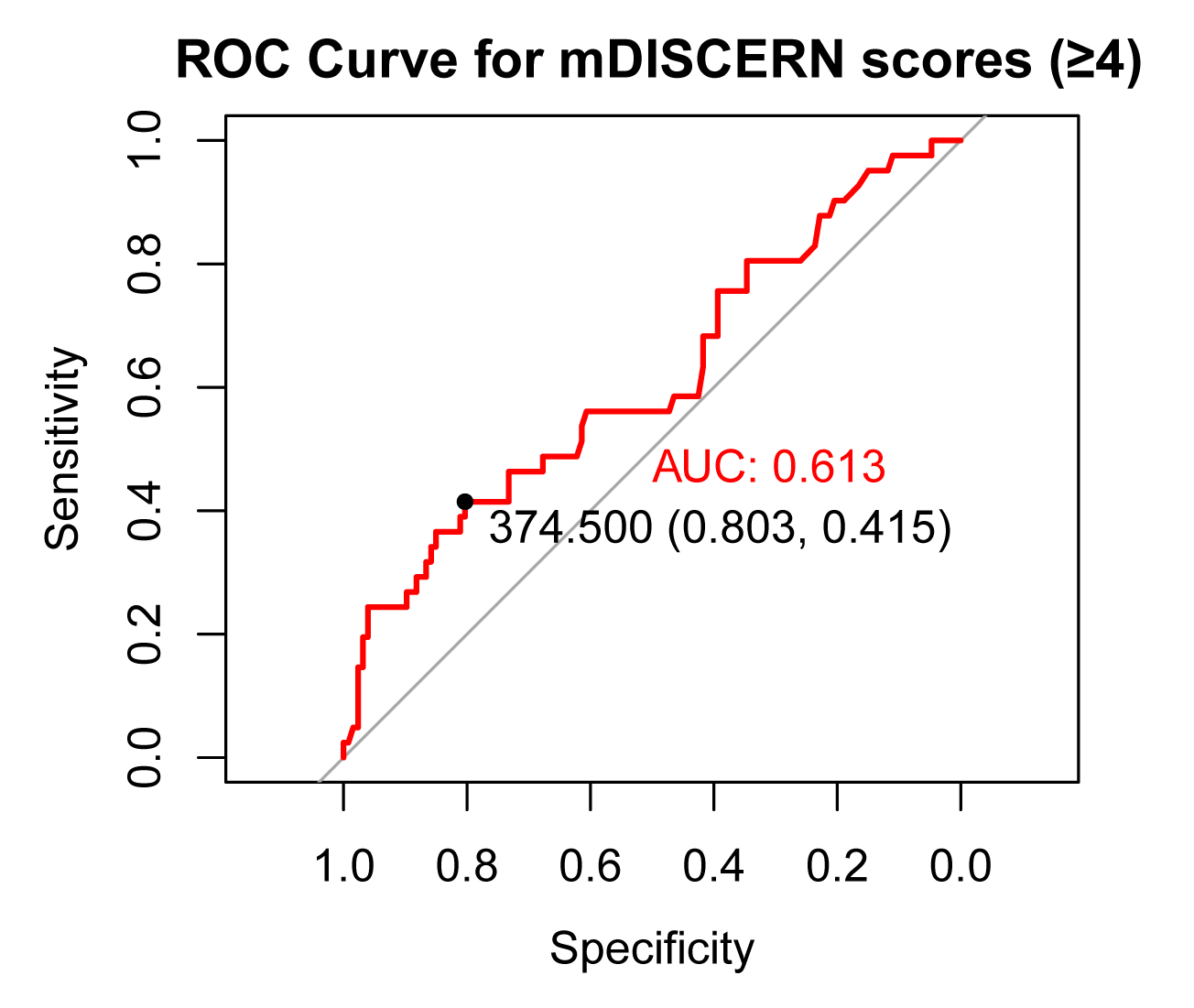

Supplement: Multimedia Appendix 6 [file formative-v8-e60033-s006.png]

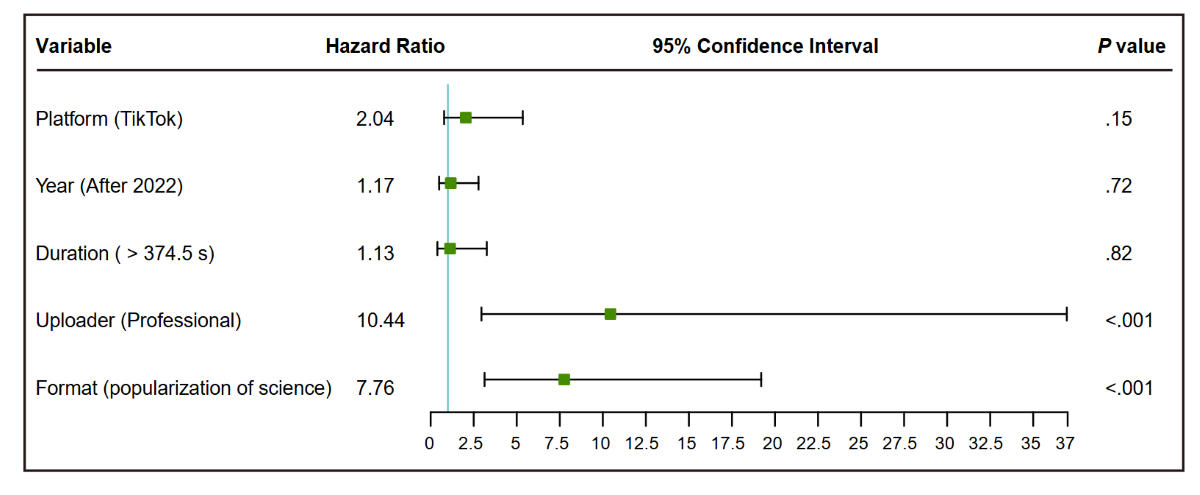

Supplement: Multimedia Appendix 9 [file formative-v8-e60033-s009.png]
