# Supplementary material for: Quality of Pancreatic Neuroendocrine Tumor Videos Available on TikTok and Bilibili: Content Analysis
Source: JMIR Form Res. 2024 Dec 11;8:e60033. doi: 10.2196/60033 (PMC11655045; doi:10.2196/60033)
Supplement: Multimedia Appendix 5 [file formative-v8-e60033-s005.docx]

| **Variable** | **Spearman correlation coefficient** | ***P* value** |
| --- | --- | --- |
| **GQS** |  |  |
| Views | 0.11 | .29 |
| Likes | −0.1 | .18 |
| Comments | −0.12 | .12 |
| Saves | −0.01 | .94 |
| Shares | 0.06 | .41 |
| Days | 0.1 | .19 |
| Duration | 0.35 | <.001 |
| **mDISCERN** |  |  |
| Views | 0.13 | .23 |
| Likes | 0.02 | .78 |
| Comments | 0.03 | .69 |
| Saves | 0.041 | .60 |
| Shares | 0.13 | .093 |
| Days | −0.02 | .76 |
| Duration | 0.15 | .04 |
