# Supplementary material for: Quality of Pancreatic Neuroendocrine Tumor Videos Available on TikTok and Bilibili: Content Analysis
Source: JMIR Form Res. 2024 Dec 11;8:e60033. doi: 10.2196/60033 (PMC11655045; doi:10.2196/60033)
Supplement: Multimedia Appendix 7 [file formative-v8-e60033-s007.docx]

| Variable | Hazard Ratio | 95% Confidence Interval | *P* value |
| --- | --- | --- | --- |
| Platform (TikTok) | 0.92 | 0.48-1.78 | .81 |
| Year (After 2022) | 0.92 | 0.46-1.84 | .81 |
| Duration ( > 374.5 s) | 2.88 | 1.18-6.99 | .02 |
| Uploader (Professional) | 6.52 | 2.34-18.16 | <.001 |
| Format (popularization of science) | 11.07 | 5.12-23.93 | <.001 |
