# Supplementary material for: Quality of Pancreatic Neuroendocrine Tumor Videos Available on TikTok and Bilibili: Content Analysis
Source: JMIR Form Res. 2024 Dec 11;8:e60033. doi: 10.2196/60033 (PMC11655045; doi:10.2196/60033)
Supplement: Multimedia Appendix 8 [file formative-v8-e60033-s008.docx]

| Variable | Hazard Ratio | 95% Confidence Interval | *P* value |
| --- | --- | --- | --- |
| Platform (TikTok) | 1.38 | 0.71-2.67 | .34 |
| Year (After 2022) | 1.39 | 0.70-2.75 | .35 |
| Duration ( > 374.5 s) | 1.36 | 0.62-2.97 | .44 |
| Uploader (Professional) | 12.44 | 3.91-39.63 | <.001 |
| Format (popularization of science) | 5.70 | 2.77-11.75 | <.001 |
